# Supplementary material for: Structural bases of IMiD selectivity that emerges by 5-hydroxythalidomide
Source: Nat Commun. 2020 Sep 14;11:4578. doi: 10.1038/s41467-020-18488-4 (PMC7490372; doi:10.1038/s41467-020-18488-4)
Supplement: Supplementary file 1 — Supplementary Information [file 41467_2020_18488_MOESM1_ESM.pdf]

Supplementary Information

Structural bases of IMiD selectivity that emerges by 5-hydroxythalidomide

Furihata et al.

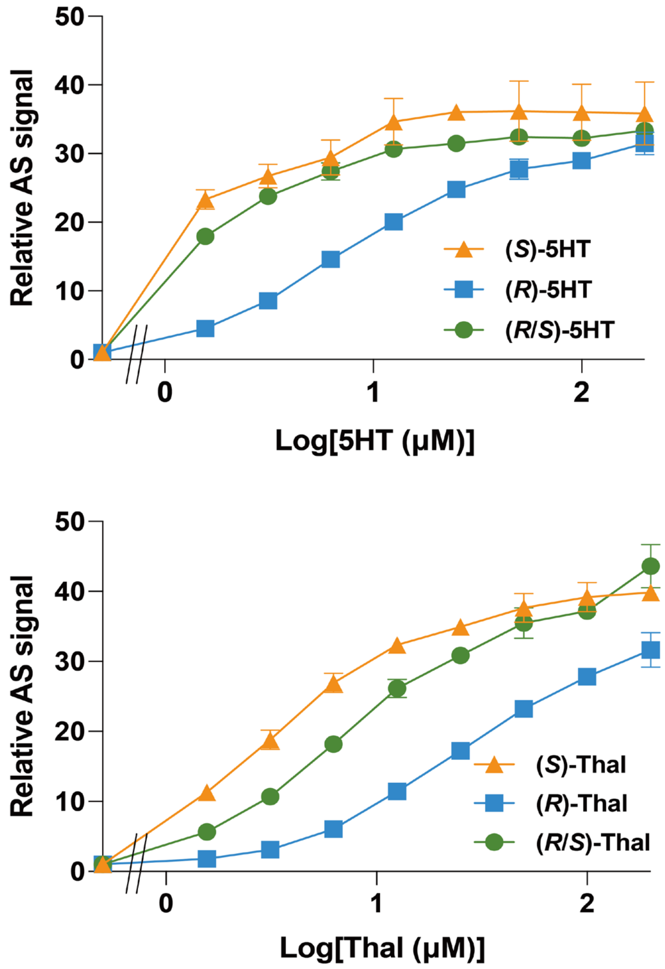

Supplementary Fig. 1 Dose-dependent interaction of SALL4 with the CRBN C366S mutant measured by the titration of racemate or each enantiomer of 5HT (upper) and thalidomide (Thal) (lower) in an AS-based assay. AS signals are expressed as the relative luminescence signal relative to the luminescence signal of DMSO, which is considered equal to one. Data are presented as mean values ± SD (n = 3 independent experiments). Source data are provided as a Source Data file.

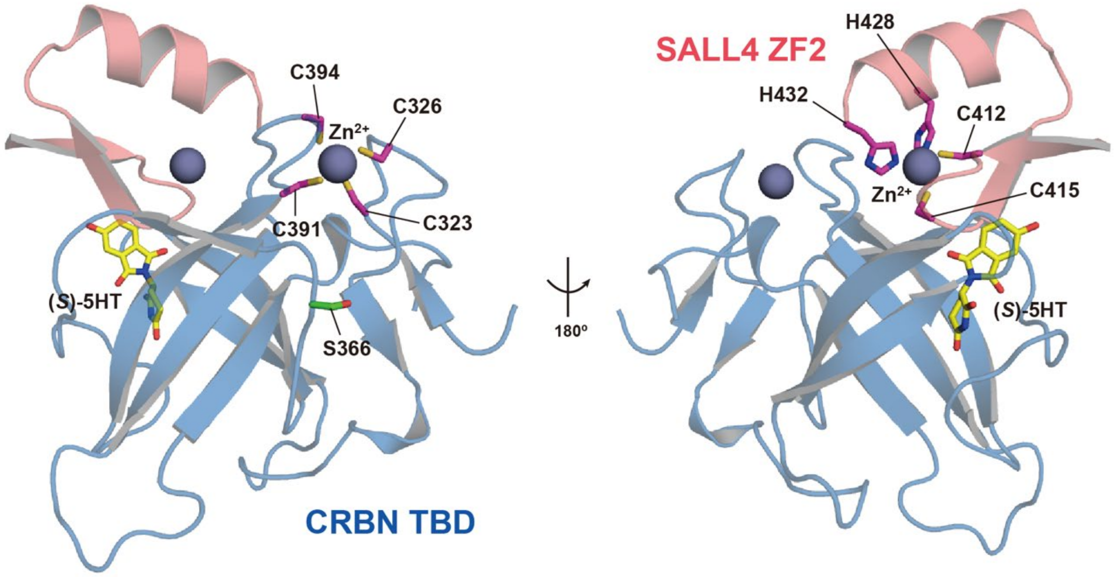

Supplementary Fig. 2 Binding modes of the zinc ions with CRBN TBD (left) and SALL4 ZF2 (right). Zinc ions (Zn^{2+}) are represented by gray spheres. Magenta sticks indicate the side chains of the Zn^{2+}-binding residues of the CRBN TBD. (*S*)-5HT is shown by a yellow stick.

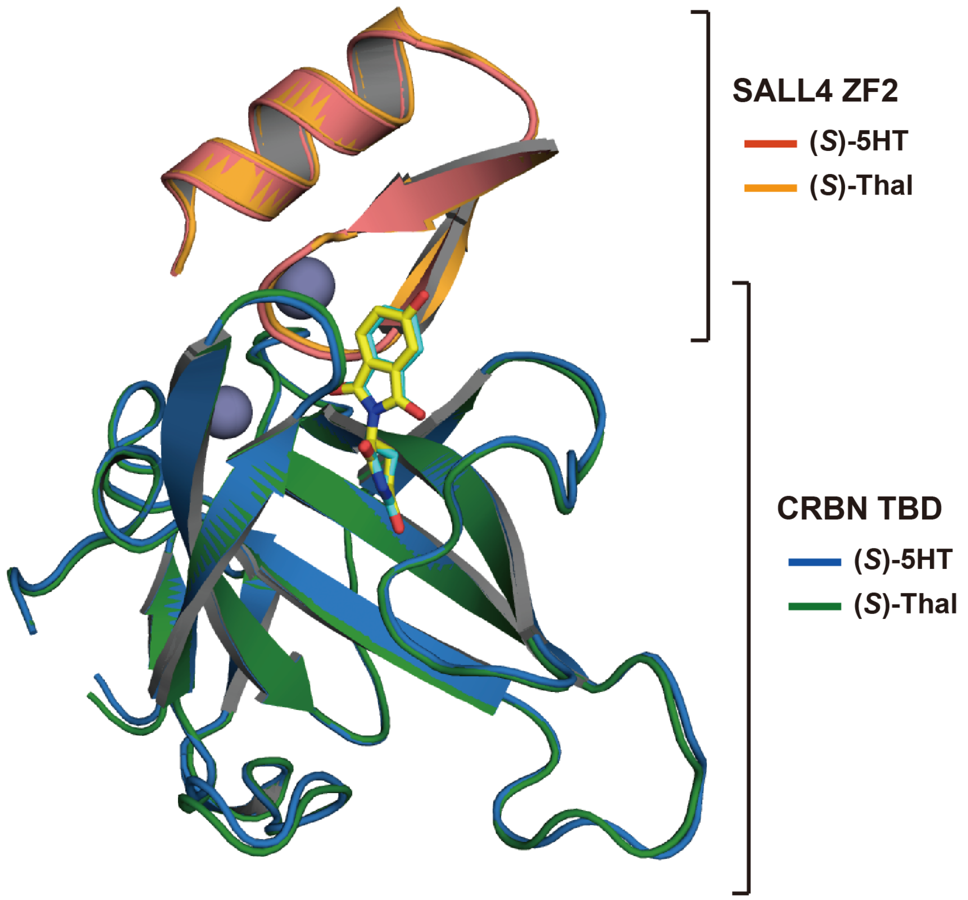

Supplementary Fig. 3 Superimposed structures of the SALL4-CRBN complex mediated by (*S*)-5HT and (*S*)-thalidomide (Thal). Gray spheres are zinc ions bound to the CRBN TBD and SALL4 ZF2. (*S*)-5HT and (*S*)-Thal are shown by yellow and cyan sticks, respectively.

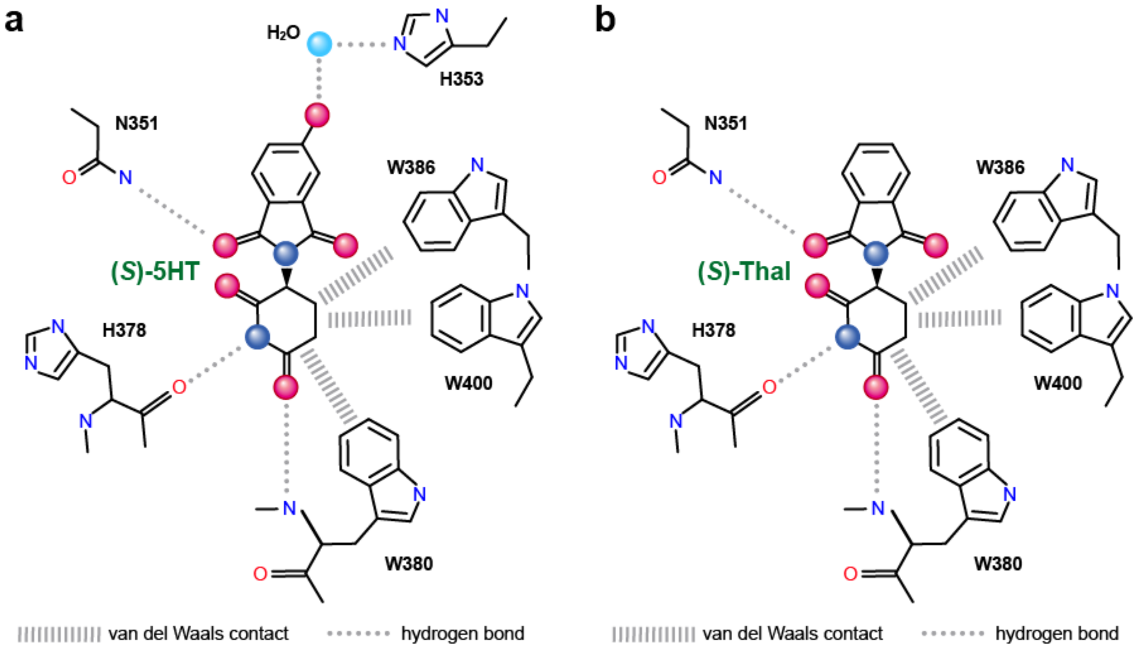

Supplementary Fig. 4 Major interactions of (*S*)-5HT and (*S*)-thalidomide (Thal) with CRBN. a, b, Magenta and blue spheres of (*S*)-5HT (**a**) and (*S*)-Thal (**b**) are oxygen and nitrogen atoms, respectively.

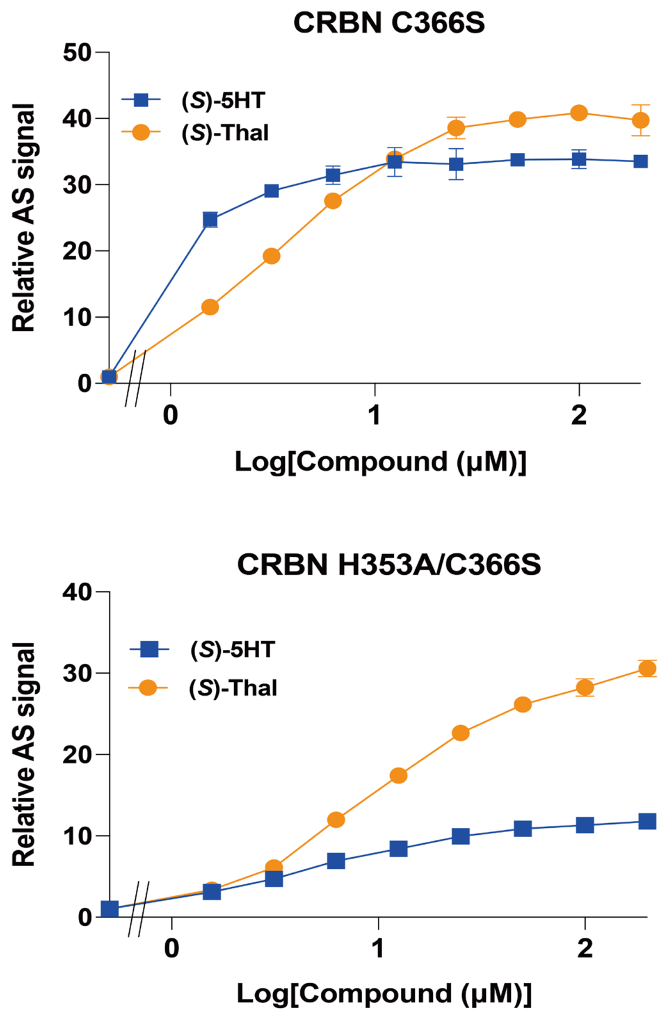

Supplementary Fig. 5 Effect of the H353A mutation in the CRBN C366S mutant on the SALL4 interaction induced by (*S*)-5HT or (*S*)-thalidomide (Thal). AS signals are expressed as the luminescence signal relative to the luminescence signal of DMSO, which is considered equal to one. Data are presented as mean values ± SD (n = 3 independent experiments). Source data are provided as a Source Data file.

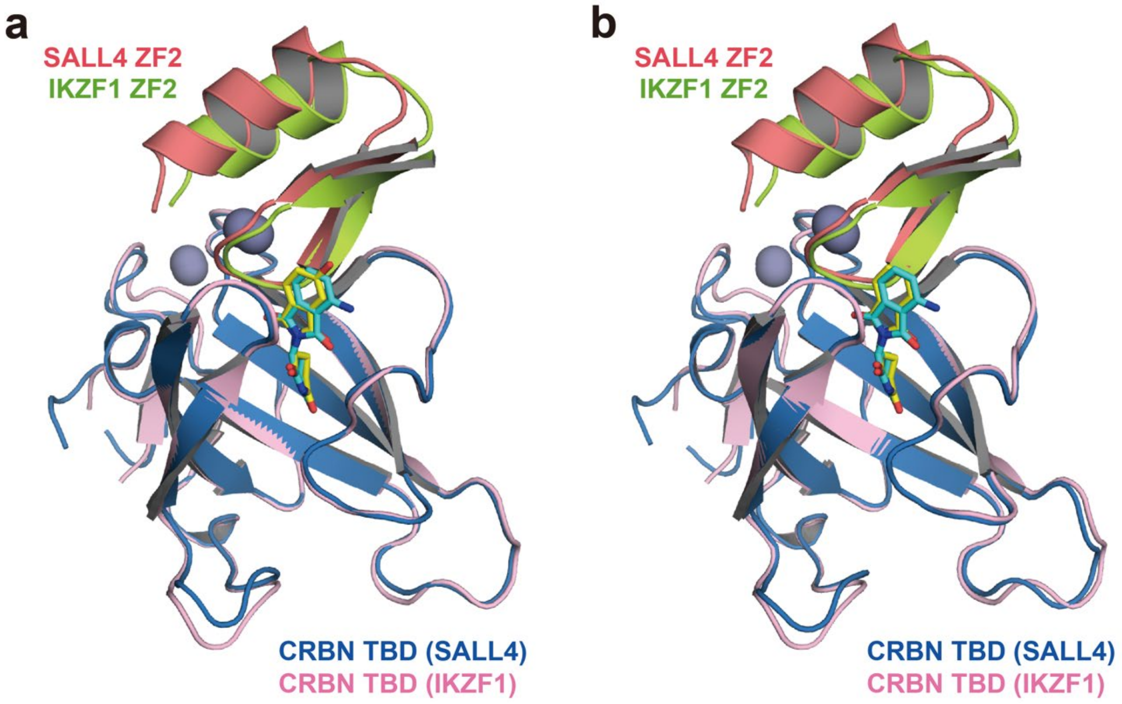

Supplementary Fig. 6 Structural comparison of SALL4 ZF2 and IKZF1 ZF2 in complex with the CRBN TBD. a, The orientation of the ZF2 domains in (*S*)-5HT-bound SALL4 and pomalidomide-bound IKZF1 relative to the CRBN TBD. (*S*)-5HT and pomalidomide are represented by yellow and cyan sticks, respectively. **b,** The orientation of the ZF2 domains of (*S*)-thalidomide (Thal)-bound SALL4 and pomalidomide-bound IKZF1 relative to the CRBN TBD. The stick models of (*S*)-Thal and pomalidomide are colored yellow and cyan, respectively. Gray spheres are zinc ions were observed in both complex structures. The structure of the IKZF1-CRBN complex with pomalidomide was generated from the coordinate data deposited in the PDB under accession number 6H0F [http://dx.doi.org/10.2210/pdb6H0F/pdb][1].

Supplementary Table 1 Data collection and refinement statistics.

	**SALL4 ZF2-CRBN TBD complex with (*S*)-5HT**	**SALL4 ZF2-CRBN TBD complex with (*S*)-Thal**
**Data collection**		
Beamline	PF AR-NE3A	PF AR-NE3A
Wavelength (Å)	1.0000	1.0000
Space group	$C222_1$	$C222_1$
Cell dimensions: *a, b, c* (Å)	83.62, 93.89, 43.68	83.62, 93.89, 43.68
Resolution (Å)	46.95–1.80 (1.84–1.80)*	43.99–1.90 (1.94–1.90)*
No. of unique reflections	16,362 (957)	11,808 (746)
R_{meas}	0.088 (1.375)	0.158 (1.310)
R_{pim}	0.024 (0.373)	0.044 (0.363)
CC(1/2)	1.000 (0.832)	0.997 (0.826)
Mean I/σ(I)	21.0 (2.3)	12.0 (2.5)
Completeness (%)	100 (100)	100 (100)
Multiplicity	13.1 (13.3)	12.9 (12.8)
**Refinement**		
Resolution (Å)	35.79–1.80	34.67–1.90
No. of reflections	16,337	11,790
R_{work}/R_{free}	0.197/0.223	0.191/0.236
No. atoms		
Protein	1,051	1,050
Ligand	20	19
Metal	2	2
Solvent	46	48
Other (SO_4)	10	–
B-factors ($Å^2$)		
Protein	37.3	31.2
Ligand	28.0	23.9
Metal	29.6	23.8
Solvent	43.9	34.1
Other (SO_4)	72.4	–
RMS deviations		
Bond lengths (Å)	0.006	0.011
Bond angles (º)	0.858	1.375
Ramachandran plot (%)		
Favoured region	98.45	97.67
Allowed region	1.55	2.33
Outliers	0.00	0.00

*Values in parentheses indicate those of the highest resolution shell.

Supplementary Reference

1. Sievers, Q. L. et al. Defining the human C2H2 zinc finger degrome targeted by thalidomide analogs through CRBN. *Science* **362**, 558 (2018).
