# Supplementary material for: Structural bases of IMiD selectivity that emerges by 5-hydroxythalidomide
Source: Nat Commun. 2020 Sep 14;11:4578. doi: 10.1038/s41467-020-18488-4 (PMC7490372; doi:10.1038/s41467-020-18488-4)
Supplement: Supplementary file 3 — Reporting Summary [file 41467_2020_18488_MOESM3_ESM.pdf]

nature research

Corresponding author(s): M. Tanokura, T. Sawasaki, T. Miyakawa
Last updated by author(s): Aug 21, 2020

Reporting Summary

Nature Research wishes to improve the reproducibility of the work that we publish. This form provides structure for consistency and transparency in reporting. For further information on Nature Research policies, see Authors & Referees and the Editorial Policy Checklist.

Statistics

For all statistical analyses, confirm that the following items are present in the figure legend, table legend, main text, or Methods section.

n/a	Confirmed	
☐	☒	The exact sample size (*n*) for each experimental group/condition, given as a discrete number and unit of measurement
☐	☒	A statement on whether measurements were taken from distinct samples or whether the same sample was measured repeatedly
☒	☐	The statistical test(s) used AND whether they are one- or two-sided<br>*Only common tests should be described solely by name; describe more complex techniques in the Methods section.*
☒	☐	A description of all covariates tested
☒	☐	A description of any assumptions or corrections, such as tests of normality and adjustment for multiple comparisons
☐	☒	A full description of the statistical parameters including central tendency (e.g. means) or other basic estimates (e.g. regression coefficient) AND variation (e.g. standard deviation) or associated estimates of uncertainty (e.g. confidence intervals)
☒	☐	For null hypothesis testing, the test statistic (e.g. *F*, *t*, *r*) with confidence intervals, effect sizes, degrees of freedom and *P* value noted<br>*Give P values as exact values whenever suitable.*
☒	☐	For Bayesian analysis, information on the choice of priors and Markov chain Monte Carlo settings
☒	☐	For hierarchical and complex designs, identification of the appropriate level for tests and full reporting of outcomes
☒	☐	Estimates of effect sizes (e.g. Cohen's *d*, Pearson's *r*), indicating how they were calculated

Our web collection on statistics for biologists contains articles on many of the points above.

Software and code

Policy information about availability of computer code

Data collection	X-ray Diffraction data, Pilatus 2M-F detector and UGUI control system with an AR-NE3A beamline at the Photo Factory; ITC data, MicroCal iTC200; AlphaScreen data, PerkinElmer Envision plate reader; and Immunoblot data, Image Quant LAS 4000mini
Data analysis	XDS (Version March 15, 2019), Aimless 0.5.17, PHENIX1-15-1, Coot 0.8.9.2, PyMol 2.0, Origin 7.0, Image J (Version 2.0.0-rc-43/1.50e)

For manuscripts utilizing custom algorithms or software that are central to the research but not yet described in published literature, software must be made available to editors/reviewers. We strongly encourage code deposition in a community repository (e.g. GitHub). See the Nature Research guidelines for submitting code & software for further information.

Data

Policy information about availability of data

All manuscripts must include a data availability statement. This statement should provide the following information, where applicable:

- Accession codes, unique identifiers, or web links for publicly available datasets
- A list of figures that have associated raw data
- A description of any restrictions on data availability

Coordinates and structure factors are available in the Protein Data Bank (PDB) under accession numbers 7BQV [http://dx.doi.org/10.2210/pdb7BQV/pdb] for the SALL4-CRBN complex with (S)-5HT and 7BQU [http://dx.doi.org/10.2210/pdb7BQU/pdb] for the SALL4-CRBN complex with (S)-thalidomide. The unprocessed scans of immunoblots are provided in a Source data file provided with this paper. Otherwise, the datasets generated and/or analysed during the current study are available from the corresponding author on reasonable request. PDB coordinates used in this study are as follows: 4TZ4 [http://dx.doi.org/10.2210/pdb4TZ4/pdb], 5YJ0 [http://dx.doi.org/10.2210/pdb5YJ0/pdb], 5YJ1 [http://dx.doi.org/10.2210/pdb5YJ1/pdb], 6H0F [http://dx.doi.org/10.2210/pdb6H0F/pdb] and 6UML [http://dx.doi.org/10.2210/pdb6UML/pdb].

Field-specific reporting

Please select the one below that is the best fit for your research. If you are not sure, read the appropriate sections before making your selection.

☒ Life sciences ☐ Behavioural & social sciences ☐ Ecological, evolutionary & environmental sciences

For a reference copy of the document with all sections, see nature.com/documents/nr-reporting-summary-flat.pdf

Life sciences study design

All studies must disclose on these points even when the disclosure is negative.

Sample size	We have chosen three independent experiments (n = 3) as a minimum size for statistics according to our previous experience in similar experimental setups. No sample-size calculation was performed.
Data exclusions	No data were excluded from the analyses.
Replication	Proteasomal degradation assays were three times independently with similar results.
Randomization	Randomization was not relevant because there is no allocation of samples/organisms/participants involved in this study.
Blinding	Investigators were not blinded to group allocation during data collection and/or analysis because there is no group allocation involved in this study.

Reporting for specific materials, systems and methods

We require information from authors about some types of materials, experimental systems and methods used in many studies. Here, indicate whether each material, system or method listed is relevant to your study. If you are not sure if a list item applies to your research, read the appropriate section before selecting a response.

Materials & experimental systems

n/a	Involved in the study	
☐	☒	Antibodies
☐	☒	Eukaryotic cell lines
☒	☐	Palaeontology
☒	☐	Animals and other organisms
☒	☐	Human research participants
☒	☐	Clinical data

Methods

n/a	Involved in the study	
☒	☐	ChIP-seq
☒	☐	Flow cytometry
☒	☐	MRI-based neuroimaging

Antibodies

Antibodies used	Epitope-tagged protein detection: Anti-FLAG mouse mAb (Sigma-Aldrich, A8592), anti-AGIA rabbit mAb (produced in our laboratory and cited to reference number 46 as the original source), anti-Myc mouse mAb (Cell Signaling Technology, 2276), and anti-DYKDDDDK mouse mAb (FUJIFILM Wako Pure Chemical Corporation, 014-22383).<br>α-Tubulin detection: Anti-α-tubulin rabbit pAb (MBL, PM054-7).<br>Biotinylated protein detection: Anti-biotin antibody (Cell Signaling Technology, 7075).
Validation	Anti-FLAG mouse mAb is an HRP-conjugated mouse monoclonal IgG1, which is recommended for detection of FLAG sequence (DYKDDDDK). The dilution ratio is 1:5,000 for WB.<br>Anti-AGIA rabbit mAb is an HRP-conjugated rabbit monoclonal IgG, which is recommended for detection of AGIA sequence (EEAAGIARP). The dilution ratio is 1:10,000 for WB.<br>Anti-Myc mouse mAb is an HRP-conjugated mouse monoclonal IgG2a, which is recommended for detection of Myc sequence (EQKLISEEDL). The dilution ratio is 1:3,000 for WB.<br>Anti-α-tubulin rabbit pAb is an HRP-conjugated rabbit polyclonal IgG, which is recommended for detection of α-tubulin. The dilution ratio is 1:10,000 for WB.<br>Anti-biotin antibody is an HRP-conjugated goat IgG, which is recommended for detection of biotinylated proteins. The dilution ratio is 1:5,000 for WB.<br>Anti-DYKDDDDK mouse mAb is a mouse monoclonal IgG2b, which is recommended for detection of FLAG sequence (DYKDDDDK). The dilution ratio is 1:2,500 for AlphaScreen.

Eukaryotic cell lines

Policy information about cell lines

Cell line source(s)	HEK293T cells were purchased from Riken BioResource Research Center (Riken BRC).
Authentication	Cell lines used were not authenticated.
Mycoplasma contamination	Cell lines used were tested negative for mycoplasma.
Commonly misidentified lines (See ICLAC register)	No commonly misidentified cell lines were used.
